# Supplementary material for: A genome‐scale yeast library with inducible expression of individual genes
Source: Mol Syst Biol. 2021 Jun 7;17(6):e10207. doi: 10.15252/msb.202110207 (PMC8182650; doi:10.15252/msb.202110207)
Supplement: Supplementary file 1 — Appendix [file MSB-17-e10207-s008.pdf]

Appendix

A genome-scale yeast library with inducible expression of individual genes

Yuko Arita[1,2,3,*], Griffin Kim[4,*], Zhijian Li[1,2,*], Helena Friesen[1], Gina Turco[4], Rebecca Y. Wang[4], Dale Climie[1,2], Matej Usaj[1,2], Manuel Hotz[4], Emily H. Stoops[4], Anastasia Baryshnikova[4], Charles Boone[1,2,3,^], David Botstein[4,^], Brenda J. Andrews[1,2,^], R. Scott McIsaac[4,^]

1. Terrence Donnelly Centre for Cellular and Biomolecular Research, University of Toronto, Toronto, Ontario M5S 3E1, Canada.

2. Department of Molecular Genetics, University of Toronto, Toronto, Ontario M5S 3E1, Canada.

3. RIKEN Centre for Sustainable Resource Science, Wako, Saitama, Japan.

4. Calico Life Sciences LLC, South San Francisco, CA 94080, USA.

*Authors contributed equally to this study.
^Corresponding authors: R. Scott McIsaac (rsm@calicolabs.com); David Botstein (botstein@calicolabs.com); Charles Boone (charles.boone@utoronto.ca); Brenda J. Andrews (brenda.andrews@utoronto.ca)

Table of Contents

Sequence information 3

Media recipes 5

Yeast Strains 7

Plasmids 9

Appendix Figure S1: Characterization of Z_3EV promoter behaviour 10

Appendix Figure S2: Gene expression responses of *ILV* and *HIS* genes with Bat1 dependence 11

Appendix Figure S3: Comparing growth and transcriptional induction for 18 YETI-NE strains 12

Appendix Figure S4: Native expression versus YETI strain growth without inducer 13

Appendix Figure S5: Z_3pr-*SHM2* is an adenine auxotroph 14

Appendix Figure S6: Identification of genes that impair growth when overexpressed 15

Appendix Figure S7: Non-essential toxic genes encode proteins that are bigger and more disordered than non-toxic genes 16

Appendix Figure S8: Comparing pooled screens to plate-based assays 17

Appendix Figure S9: Conditional growth of Z_3EV-driven essential genes is not reversible in 16 of 24 strains tested 18

Appendix Figure S10: Reversibility analysis for 19 strains that impair growth when overexpressed 19

Appendix Figure S11: Reversibility analysis of Z_3EV and Z_3EB42 using mNeonGreen reporter 20

Sequence information

p5820, Z_3 promoter sequence (6 binding sites)
The sequence of Z_3 (6 binding sites) promoter is shown below. Modified UAS_{GAL} for Z_3EV binding is highlighted in green. XbaI and NotI restriction sites are colored with gray. A Gal4 binding site outside of XbaI and NotI restriction sites was also removed.

TTATATTGAATTTTCAAAAATTCTTACTTTTTTTTTGGATGGACGCAAAGAAGTTTAATAATCATATTACATGGCATTACCACCATAT
ACATATCCATATACATATCCATATCTAATCTTACTTATATGTTGTGGAAATGTAAAGAGCCCCATTATCTTAGCCTAAAAAAACCTT
CTCTTTGGAACTTTCAGTAATACGCTTAACTGCTCATTGCTATATTGAAGTGCGGCCGCGTGGGCGTGCGTGGGCGGGCGTGG
GCGTGCGTGGGCGGGCGTGGGCGTGCGTGGGCGTCTAGACCGTGCGTCCTCGTCTTCACCGGTCGCGTTCCTGAAACGCAG
ATGTGCCTCGCGCCGCACTGCTAGCAACAATAAAGATTCTACAATACTAGCTTTTATGGTTATGAAGAGGAAAAATTGGCAGTAA
CCTGGCCCCACAAACCTTCAAATTAACGAATCAAATTAACAACCATAGGATGATAATGCGATTAGTTTTTTAGCCTTATTTCTGGG
GTAATTAATCAGCGAAGCGATGATTTTTGATCTATTAACAGATATATAAATGGAAAAGCTGCATAACCACTTTAACTAATACTTTC
AACATTTTCAGTTTGTATTACTTCTTATTCAAATGTCATAAAAGTATCAACAAAAAATTGTTAATATACCTCTATACTTTAACGTCAA
GGAGAAAAAACTATA

p7418, Z_3 promoter sequence (2 binding sites)
TTATATTGAATTTTCAAAAATTCTTACTTTTTTTTTGGATGGACGCAAAGAAGTTTAATAATCATATTACATGGCATTACCACCATAT
ACATATCCATATACATATCCATATCTAATCTTACTTATATGTTGTGGAAATGTAAAGAGCCCCATTATCTTAGCCTAAAAAAACCTT
CTCTTTGGAACTTTCAGTAATACGCTTAACTGCTCATTGCTATATTGAAGTGCGGCCGCGTGGGCGTGCGTGGGCGGTCTAGAC
CGTGCGTCCTCGTCTTCACCGGTCGCGTTCCTGAAACGCAGATGTGCCTCGCGCCGCACTGCTAGCAACAATAAAGATTCTAC
AATACTAGCTTTTATGGTTATGAAGAGGAAAAATTGGCAGTAACCTGGCCCCACAAACCTTCAAATTAACGAATCAAATTAACAA
CCATAGGATGATAATGCGATTAGTTTTTTAGCCTTATTTCTGGGGTAATTAATCAGCGAAGCGATGATTTTTGATCTATTAACAGA
TATATAAATGGAAAAGCTGCATAACCACTTTAACTAATACTTTCAACATTTTCAGTTTGTATTACTTCTTATTCAAATGTCATAAAA
GTATCAACAAAAAATTGTTAATATACCTCTATACTTTAACGTCAAGGAGAAAAAACTATA

p7460, Z_3 (6 binding sites)-URS1 promoter sequence
Modified UAS_{GAL} forATF binding is highlighted in green. XbaI and NotI restriction sites are colored with gray. URS1 sequence of CAR1 is highlighted in blue.

TTATATTGAATTTTCAAAAATTCTTACTTTTTTTTTGGATGGACGCAAAGAAGTTTAATAATCATATTACATGGCATTACCACCATAT
ACATATCCATATACATATCCATATCTAATCTTACTTATATGTTGTGGAAATGTAAAGAGCCCCATTATCTTAGCCTAAAAAAACCTT
CTCTTTGGAACTTTCAGTAATACGCTTAACTGCTCATTGCTATATTGAAGTGCGGCCGCGTGGGCGTGCGTGGGCGGGCGTGG
GCGTGCGTGGGCGGGCGTGGGCGTGCGTGGGCGTCTAGACCGTGCGTCCTCGTCTTCACCGGTCGCGTTCCTGAAACGCAG
ATGTGCCTCGCGCCGCACTGCTAGCAACAATAAAGATTCTACAATACTAGCTTTTATGGTTATGAAGAGGAAAAATTGGCAGTAA
CCTGGCCCCACAAACCTTCAAATTAACGAATCAAATTAACAACCATAGGATGATAATGCGATTAGTTTTTTAGCCTTATTTCTGGG
GTAATTAATCAGCGAAGCGATGATTTTTGATCTATTAACAGATATATAAATGGAAAAGCTGCATAACCACTTTAACTAATACTTTC
AACATTTTCAGTTTGTATTACTTCTTATTCAAATGTCATAAAAGTATCAACAAAAAATTGTTAATATACCTCTATATCAACAGCGCA
TCGCCGCTCGCTGAATTTTTCACTTAGCGGTAGCCGCCGAGGGGTCTAAAGAGTATATAAGCAGAGCTTGCGGCCCACTTTCTA
TCAAGATCTAAGACTGTTTCTCTTCTCTTGGTCTGTATATGTTTTCTCAAAGTTAGCAGAAACAACAACAACAACTATATCAATAA
CAATAACTACTATCAAGCTTTAACGTCAAGGAGAAAAAACTATA

p7288, Z_3 (2 binding sites)-URS1 promoter sequence
Modified UAS_{GAL} for ATF binding is highlighted in green. XbaI and NotI restriction sites are colored with gray. URS1 sequence of CAR1 is highlighted in blue.

TTATATTGAATTTTCAAAAATTCTTACTTTTTTTTTGGATGGACGCAAAGAAGTTTAATAATCATATTACATGGCATTACCACCATAT
ACATATCCATATACATATCCATATCTAATCTTACTTATATGTTGTGGAAATGTAAAGAGCCCCATTATCTTAGCCTAAAAAAACCTT
CTCTTTGGAACTTTCAGTAATACGCTTAACTGCTCATTGCTATATTGAAGTGCGGCCGCGTGGGCGTGCGTGGGCGGTCTAGAC
CGTGCGTCCTCGTCTTCACCGGTCGCGTTCCTGAAACGCAGATGTGCCTCGCGCCGCACTGCTAGCAACAATAAAGATTCTAC
AATACTAGCTTTTATGGTTATGAAGAGGAAAAATTGGCAGTAACCTGGCCCCACAAACCTTCAAATTAACGAATCAAATTAACAA
CCATAGGATGATAATGCGATTAGTTTTTTAGCCTTATTTCTGGGGTAATTAATCAGCGAAGCGATGATTTTTGATCTATTAACAGA
TATATAAATGGAAAAGCTGCATAACCACTTTAACTAATACTTTCAACATTTTCAGTTTGTATTACTTCTTATTCAAATGTCATAAAA
GTATCAACAAAAAATTGTTAATATACCTCTATATCAACAGCGCATCGCCGCTCGCTGAATTTTTCACTTAGCGGTAGCCGCCGAG

GGGTCTAAAGAGTATATAAGCAGAGCTTGCGGCCCACTTTCTATCAAGATCTAAGACTGTTTCTCTTCTCTTGGTCTGTATATGT
TTTCTCAAAGTTAGCAGAAACAACAACAACAACTATATCAATAACAATAACTACTATCAAGCTTTAACGTCAAGGAGAAAAAACTA
TA

Z_3EB42 sequence

The *ACT1* promoter is shown in blue; the Z_3 DNA binding domain is shown in green; the human Estrogen Receptor is shown in magenta; the B42 activation domain is in red; the *ENO2* terminator is in orange. The transcription factor was integrated 300 bp downstream of functional *HAP1*. 300 bp of downstream sequence of *HAP1* was also added downstream of the Z_3EB42 transcription factor.

GCCTCTACCTTGCAGACCCATATAATATAATAACTAAATAAGTAAATAAGACACACGCGAGAACATATATACACAATTACAGTAAC
AATAACAAGAGGACAGATACTACCAAAATGTGTGGGGAAGCGGGTAAGCTGCCACAGCAATTAATGCACAACATTTAACCTACA
TTCTTCCTTATCGGATCCTCAAAACCCTTAAAAACATATGCCTCACCCTAACATATTTTCCAATTAACCCTCAATATTTCTCTGTCA
CCCGGCCTCTATTTTCCATTTTCTTCTTTACCCGCCACGCGTTTTTTTCTTTCAAATTTTTTTCTTCCTTCTTCTTTTTCTTCCACGT
CCTCTTGCATAAATAAATAAACCGTTTTGAAACCAAACTCGCCTCTCTCTCTCCTTTTTGAAATATTTTTGGGTTTGTTTGATCCTT
TCCTTCCCAATCTCTCTTGTTTAATATATATTCATTTATATCACGCTCTCTTTTTATCTTCCTTTTTTTCCTCTCTCTTGTATTCTTCC
TTCCCCTTTCTACTCAAACCAAGAAGAAAAAGAAAAGGTCAATCTTTGTTAAAGAATAGGATCTTCTACTACATCAGCTTTTAGAT
TTTTCACGCTTACTGCTTTTTTCTTCCCAAGATCGAAAATTTACTGAATTAACAGGGCCCCCCCTCGAGGTCGACGGTATCGATA
AGCTTGAAGCAAGCCTCCTGAAAGATGGGTACCCGCCCATATGCTTGCCCTGTCGAGTCCTGCGATCGCCGCTTTTCTCGCTC
GGATGAGCTTACCCGCCATATCCGCATCCATACCGGTCAGAAGCCCTTCCAGTGTCGAATCTGCATGCGTAACTTCAGTCGTAG
TGACCACCTTACCACCCACATCCGCACCCACACAGGCGAGAAGCCTTTTGCCTGTGACATTTGTGGGAGGAAGTTTGCCAGGA
GTGATGAACGCAAGAGGCATACCAAAATCCATACAGGTGGCGGAGGCACACCTGCAGCTGCGTCGACTCTAGAGGATCCATCT
GCTGGAGACATGAGAGCTGCCAACCTTTGGCCAAGCCCGCTCATGATCAAACGCTCTAAGAAGAACAGCCTGGCCTTGTCCCT
GACGGCCGACCAGATGGTCAGTGCCTTGTTGGATGCTGAGCCCCCCATACTCTATTCCGAGTATGATCCTACCAGACCCTTCA
GTGAAGCTTCGATGATGGGCTTACTGACCAACCTGGCAGACAGGGAGCTGGTTCACATGATCAACTGGGCGAAGAGGGTGCC
AGGCTTTGTGGATTTGACCCTCCATGATCAGGTCCACCTTCTAGAATGTGCCTGGCTAGAGATCCTGATGATTGGTCTCGTCTG
GCGCTCCATGGAGCACCCAGTGAAGCTACTGTTTGCTCCTAACTTGCTCTTGGACAGGAACCAGGGAAAATGTGTAGAGGGCA
TGGTGGAGATCTTCGACATGCTGCTGGCTACATCATCTCGGTTCCGCATGATGAATCTGCAGGGAGAGGAGTTTGTGTGCCTC
AAATCTATTATTTTGCTTAATTCTGGAGTGTACACATTTCTGTCCAGCACCCTGAAGTCTCTGGAAGAGAAGGACCATATCCACC
GAGTCCTGGACAAGATCACAGACACTTTGATCCACCTGATGGCCAAGGCAGGCCTGACCCTGCAGCAGCAGCACCAGCGGCT
GGCCCAGCTCCTCCTCATCCTCTCCCACATCAGGCACATGAGTAACAAAGGCATGGAGCATCTGTACAGCATGAAGTGCAAGA
ACGTGGTGCCCCTCTATGACCTGCTGCTGGAGATGCTGGACGCCCACCGCCTACATGCGCCCACTAGCCGTGGAGGGGCATC
CGTGGAGGAGACGGACCAAAGCCACTTGGCCACTGCGGGCTCTACTTCATCGGGTATCAATAAAGAcATCGAGGAGTGCAATG
CCATCATTGAGCAGTTTATCGACTACCTGCGCACCGGACAGGAGATGCCGATGGAAATGGCGGATCAGGCGATTAACGTGGTG
CCGGGCATGACGCCGAAAACCATTCTTCACGCCGGGCCGCCGATCCAGCCTGACTGGCTGAAATCGAATGGTTTTCATGAAAT
TGAAGCGGATGTTAACGATACCAGCCTCTTGCTGAGTGGAGATGCCTCCAAGCTTTAAATCCCCGCGTGCTTGGCCGGCCGTA
GTGCTTTTAACTAAGAATTATTAGTCTTTTCTGCTTATTTTTTCATCATAGTTTAGAACACTTTATATTAACGAATAGTTTATGAATC
TATTTAGGTTTAAAAATTGATACAGTTTTATAAGTTACTTTTTCAAAGACTCGTGCTGTCTATTGCATAATGCACTGGAAGGGGAA
AAAAAAGGTGCACACGCGTGGCTTTTTCTTGAATTTGCAGTTTGAAAAATAACTACATGGATGATAAGAAAACATGGAGTACAGT
CACTTTGAGAACCTTCAATCAGCTGGTAACGTCTTCGTTAATTGGATACTCAAAAAAGATGGATAGCATGAATCACAAGATGGAA
GGAAATGCGGGCCACGACCACAGTGATATGCATATGGGAGATGGAGATGATACCTGTTCGATGAATATGCTATTTTCGTGGTCA
TACAAGAATACGTGTGTCGTCTTTGAATGGTGGCATATCAAGACCCTGCCTGGACTGAT

Media recipes

Z3-E and Z3-NE Diploid Maintenance Medium

YPD+ClonNAT

For 1L

- In 900 mL water, add
  10 g Yeast Extract
  20 g Bacto Peptone
  20 g Agar
- autoclave
- Add 100 mL 20% glucose (w/v)
- Mix well and cool to ~60°C
- Add 1 mL 1000X ClonNAT (100mg/mL; final concentration 100 μg/mL)

Sporulation Medium

For 1L

- 10 g potassium acetate
- 1 g yeast extract
- 20 g bacto agar to 1L water
- After autoclaving, cool to 50°C
- 2.5 mL of 20% glucose (w/v)
- 1.67 mL 100X histidine (8.56 g/L; final concentration ~14 mg/L)
- 166.7 μL of 1000X G418 stock solution (300 mg/mL; final concentration 50 μg/L)

Z3-E Haploid Selection Medium (SC-arg-his-lys-ura)+YNB+glucose+msg+canavanine+thialysine+ClonNAT+β-estradiol

For 5L

- In 3L water, add
  8.5g YNB w/o Amino Acids w/o Ammonium Sulfate
  100g agar
  milliQ water up to 3.5L
- Autoclave, cool to 50°C, and add
  500mL 10X SC-arg-his-lys-ura (manufacturer's recommendation)
  500mL 20% glucose (w/v)
  500mL 10X msg (760 mM; final concentration 76mM)
  5mL 1000X canavanine (60 mg/mL; final concentration 60 μg/mL)
  2.5mL 2000X thialysine (100 mg/mL; final concentration 100 μg/mL)
  5mL 1000X ClonNAT (100mg/mL; final concentration 100 μg/mL)
  5mL 1000X β-estradiol (1000X of final concentration assayed, in ethanol)

Z3-NE Haploid Maintenance Medium
SC+YNB+glucose+msg+ClonNAT

For 5L

- In 3L water, add
  8.5g YNB w/o Amino Acids w/o Ammonium Sulfate
  100g agar
  milliQ water up to 3.5L
- Autoclave, cool to 50°C, and add
  500mL 10X SC (manufacturer's recommendation)
  500mL 20% glucose (w/v)
  500mL 10X msg (760 mM; final concentration 76mM)
  5mL 1000X ClonNAT (100mg/mL; final concentration 100 μg/mL)

<u>Z3-NE Haploid SC Medium</u>
SC+YNB+glucose+msg+ClonNAT+β-estradiol
For 5L

- In 3L water, add
  8.5g YNB w/o Amino Acids w/o Ammonium Sulfate
  100g agar
  milliQ water up to 3.5L
- Autoclave, cool to 50°C, and add
  500mL 10X SC (manufacturer's recommendation)
  500mL 20% glucose (w/v)
  500mL 10X msg (760 mM; final concentration 76mM)
  5mL 1000X ClonNAT (100mg/mL; final concentration 100 μg/mL)
  5mL 1000X β-estradiol (1000X of final concentration assayed, in ethanol)

<u>Z3-NE Haploid Minimal Medium</u>
YNB+glucose+msg+ClonNAT+β-estradiol
For 5L

- In 3.5L water, add
  8.5g YNB w/o Amino Acids w/o Ammonium Sulfate
  100g agar
  milliQ water up to 4L
- Autoclave, cool to 50°C, and add
  500mL 20% glucose (w/v)
  500mL 10X msg (760 mM; final concentration 76mM)
  5mL 1000X ClonNAT (100mg/mL; final concentration 100 μg/mL)
  5mL 1000X β-estradiol (1000X of final concentration assayed, in ethanol)

<u>10X SC solution (for preparation of 1L)</u>

- Add 20 g of SC mix (Sunrise Cat# 1300-030) into a beaker with stir bar
- Add 900 mL of water
- Stir until dissolved (heat liquid up to 60°C to dissolve)
- Transfer into a graduated cylinder, and add water to 1000 mL
- Filter sterilize using 1000 mL stericup

<u>10X SC-arg-his-lys-ura solution (for preparation of 1L)</u>

- Add 16.6 g of SC-arg-his-lys-ura mix (Sunrise Cat# 6103-030) into a beaker with stir bar
- Add 900 mL of water
- Stir until dissolved (heat liquid up to 60°C to dissolve)
- Transfer into a graduated cylinder, and add water to 1000 mL
- Filter sterilize using 1000 mL stericup

Yeast Strains

Strain	Genotype	Source
RCY1972	MAT**a** his3Δ1 HAP1+	Gift from Amy Caudy
BY6442	MATα URA3::Z_3pr-ROF1 HAP1+::natMX::pACT1-Z_3EV-ENO2term ura3Δ0 can1Δ::STE2pr-Sphis5 his3Δ1 lyp1Δ0	this study
DBY12394	MAT**a** ura3Δ0 leu2Δ0 pr-ACT1-Z_3EV-NatMX	McIsaac et al. (2013)
Y7092	MAT**a** can1Δ::STE2pr-Sphis5 lyp1Δ his3Δ1 leu2Δ0 ura3Δ0 met15Δ0	Costanzo et al. (2010)
Y14851	MAT**a** HAP1+::natMX::ACT1pr-Z_3EV-ENO2term ura3Δ0 can1ΔSTE2pr-Sphis5 his3Δ1 lyp1Δ	this study
Y14537	MATα ura3Δ0 his3Δ1 HAP1+	this study
Y14789	MAT**a**/α HAP1+::natMX::ACT1pr-Z_3EV-ENO2term/HAP1+ ura3Δ0/ura3Δ0 can1Δ::STE2pr-Sphis5/CAN1+ his3Δ1/ his3Δ1 lyp1Δ/LYP1+	this study
Y15090	MAT**a**/α HAP1-natMX-ACT1pr-Z3EV-ENO2term/HAP1 ura3Δ0/URA3 can1Δ::STE2pr-Sphis5/CAN1 his3Δ1/his3Δ1 lyp1Δ/LYP1	this study
Y15292	MAT**a** HAP1+::natMX::ACT1pr-Z_3EV-ENO2term ura3Δ0 can1ΔSTE2pr-Sphis5 his3Δ1 lyp1Δ hoΔ::URA3::Z_3pr(6 binding sites)-GFP	this study
Y15260	MAT**a**/α HAP1+::natMX-pACT1-Z_3EB42ENO2term /HAP1+ his3Δ1/his3Δ1 ura3Δ0/ura3Δ0 lyp1Δ/LYP1 can1ΔSte2pr-Sphis5/CAN1 leu2Δ/leu2Δ URA3-Z_3pr(2 binding sites)-CAR1URS1-PBR1	this study
Y15474	MAT**a**/α HAP1+::natMX-pACT1-Z_3EB42-ENO2term /HAP1+ his3Δ1/his3Δ1 ura3Δ0/ura3Δ0 lyp1Δ/LYP1 can1ΔSte2pr-Sphis5/CAN1 leu2Δ/leu2Δ URA3-Z_3pr(2 binding sites)-CAR1URS1-RAD53	this study
Y15390	MAT**a**/α HAP1+::natMX-pACT1-Z_3EB42-ENO2term /HAP1+ his3Δ1/his3Δ1 ura3Δ0/ura3Δ0 lyp1Δ/LYP1 can1ΔSte2pr-Sphis5/CAN1 leu2Δ/leu2Δ URA3-Z_3pr(2 binding sites)-CAR1URS1-CIA2	this study
Y15475	MAT**a**/α HAP1+::natMX-pACT1-Z_3EB42-ENO2term /HAP1+ his3Δ1/his3Δ1 ura3Δ0/ura3Δ0 lyp1Δ/LYP1 can1ΔSte2pr-Sphis5/CAN1 leu2Δ/leu2Δ URA3-Z_3pr(2 binding sites)-CAR1URS1-IPL1	this study
Y15476	MAT**a**/α HAP1+::natMX-pACT1-Z_3EB42-ENO2term /HAP1+ his3Δ1/his3Δ1 ura3Δ0/ura3Δ0 lyp1Δ/LYP1 can1ΔSte2pr-Sphis5/CAN1 leu2Δ/leu2Δ URA3-Z_3pr(2 binding sites)-CAR1URS1-HYP2	this study
Y15477	MAT**a** HAP1+::natMX::ACT1pr-Z_3EV-ENO2term can1ΔSTE2pr-Sphis5 his3Δ1 lyp1Δ URA3+	this study
Y15483	MAT**a** HAP1+ can1ΔSTE2pr-Sphis5 his3Δ1 lyp1Δ ura3Δ hoΔ::URA3::Z_3pr(6 binding sites)-GFPURA3+	this study
Y15478	MAT**a**/α HAP1+::natMX::ACT1pr-Z_3EV-ENO2term/HAP1+ ura3Δ0/ura3Δ0 can1ΔSTE2pr-Sphis5/CAN1+ his3Δ1/ his3Δ1 lyp1Δ/LYP1+ URA3-Z_3pr(2 binding sites)-RAD53	this study
Y15479	MAT**a**/α HAP1+::natMX::ACT1pr-Z_3EV-ENO2term/HAP1+ ura3Δ0/ura3Δ0 can1ΔSTE2pr-Sphis5/CAN1+ his3Δ1/ his3Δ1 lyp1Δ/LYP1+ URA3-Z_3pr(4 binding sites)-RAD53	this study
cDBY0776	MAT**a** HAP1+ shm2Δ::kanMX	this study
cDBY0777	MAT**a** HAP1+ shm2Δ::kanMX	this study

cDBY0778	*MATα HAP1+ shm2Δ::kanMX*	this study
cDBY0779	MAT*α HAP1+ shm2Δ::kanMX*	this study
Y15513	*MAT***a** *HAP1+*::*natMX*::*ACT1*pr-Z_3EV-*ENO2term ura3Δ0 can1ΔSTE2*pr-*Sphis5 his3Δ1 lyp1Δ hoΔ*::*URA3*-Z_3pr*(6 binding sites)*-mNeonGreen	this study
Y15515	*MAT***a** *HAP1+*::*natMX*::*ACT1*pr-Z_3EV-*ENO2term ura3Δ0 can1ΔSTE2*pr-*Sphis5 his3Δ1 lyp1Δ hoΔ*::*URA3*-Z_3pr*(2 binding sites)*-mNeonGreen	this study
Y15516	*MAT***a** *HAP1+*::*natMX*::*ACT1*pr-Z_3EV-*ENO2term ura3Δ0 can1ΔSTE2*pr-*Sphis5 his3Δ1 lyp1Δ hoΔ*::*URA3*-Z_3pr*(6 binding sites-CAR1URS1)*-mNeonGreen	this study
Y15517	*MAT***a** *HAP1+*::*natMX*::*ACT1*pr-Z_3EV-*ENO2term ura3Δ0 can1ΔSTE2*pr-*Sphis5 his3Δ1 lyp1Δ* hoΔ::*URA3*-Z_3pr*(2 binding sites-CAR1URS1)*-mNeonGreen	this study
Y15518	*MAT***a** *HAP1+*::*natMX*::*ACT1*pr-Z_3EB42-*ENO2term ura3Δ0 can1ΔSTE2*pr-*Sphis5 his3Δ1 lyp1Δ hoΔ*::*URA3*-Z_3pr*(6 binding sites)*-mNeonGreen	this study
Y15520	*MAT***a** *HAP1+*::*natMX*::*ACT1*pr-Z_3EB42-*ENO2term ura3Δ0 can1ΔSTE2*pr-*Sphis5 his3Δ1 lyp1Δ hoΔ*::*URA3*-Z_3pr*(2 binding sites)*-mNeonGreen	this study
Y15521	*MAT***a** *HAP1+*::*natMX*::*ACT1*pr-Z_3EB42-*ENO2term ura3Δ0 can1ΔSTE2*pr-*Sphis5 his3Δ1 lyp1Δ hoΔ*::*URA3*-Z_3pr*(6 binding sites-CAR1URS1)*-mNeonGreen	this study
Y15522	*MAT***a** *HAP1+*::*natMX*::*ACT1*pr-Z_3EB42-*ENO2term ura3Δ0 can1ΔSTE2*pr-*Sphis5 his3Δ1 lyp1Δ hoΔ*::*URA3*-Z_3pr*(2 binding sites-CAR1URS1)*-mNeonGreen	this study

Plasmids

Plasmid	Description	Source
pRB3460	Original promoter that is inducible by Z_3EV (Z_3pr); contains *kanMX*	McIsaac et al. (2013)
p5820	Template for PCR targeting; contains *URA3* upstream of Z_3pr	this study
p7418	URA3-Z_3pr (2 binding sites)	this study
p7288	URA3-Z_3pr (CAR1 URS + 2 Zif268 Binding Sites)	this study
p7460	URA3-Z_3pr (CAR1 URS + 6 Zif268 Binding Sites)	this study

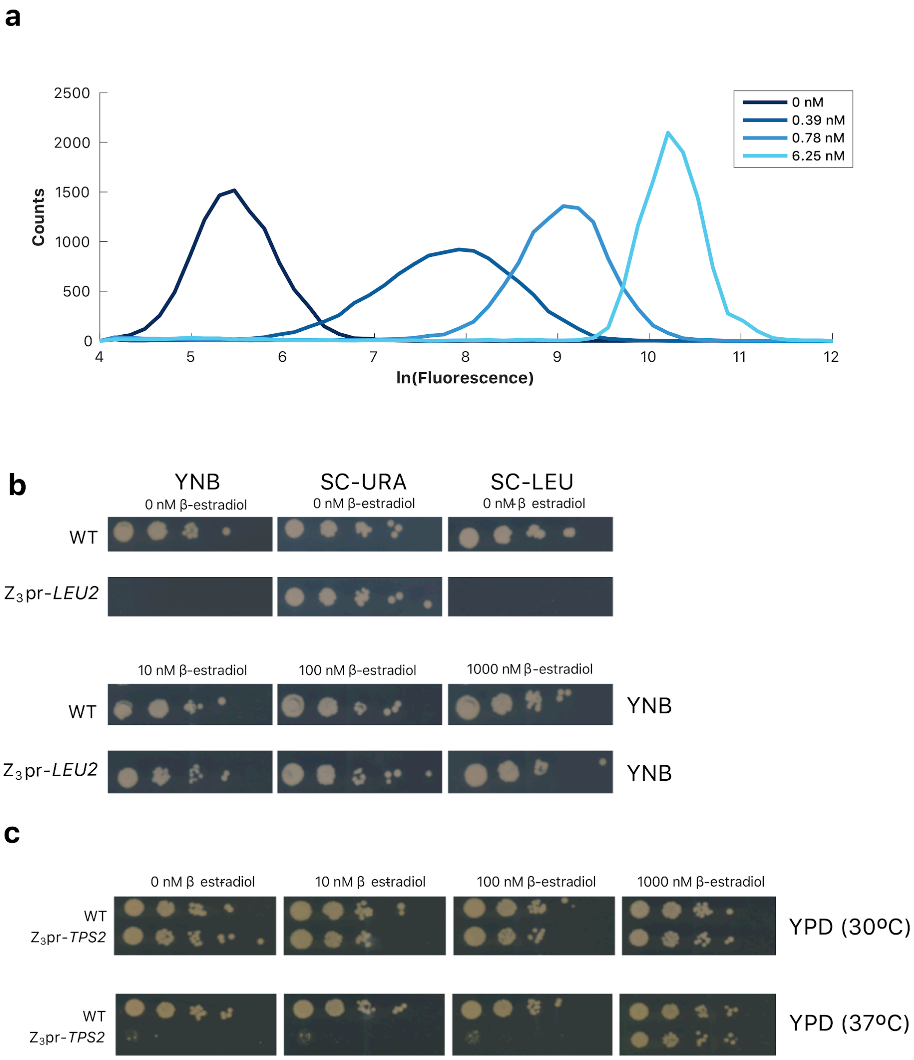

Appendix Figure S1: Characterization of Z_3EV promoter behaviour

A. Single-cell flow cytometry measurements of GFP induction expression from the Z_3pr promoter at multiple doses of β-estradiol (strain Y15292). Each distribution contains fluorescence measurements from ~10,000 cells. **B.** Characterization of Z_3pr-*LEU2* strain. Tenfold serial dilutions of WT (Y15477) and the YETI-NE strain of *LEU2* are shown. The top row shows plates with no β-estradiol. Plates were incubated at 30°C for two days. **C.** Characterization Z_3pr-*TPS2* strain**.** Y15477 and the YETI-NE strain of *TPS2* were spotted and grown at 30°C and 37°C as indicated on YPD with no β-estradiol or increasing concentrations of β-estradiol.

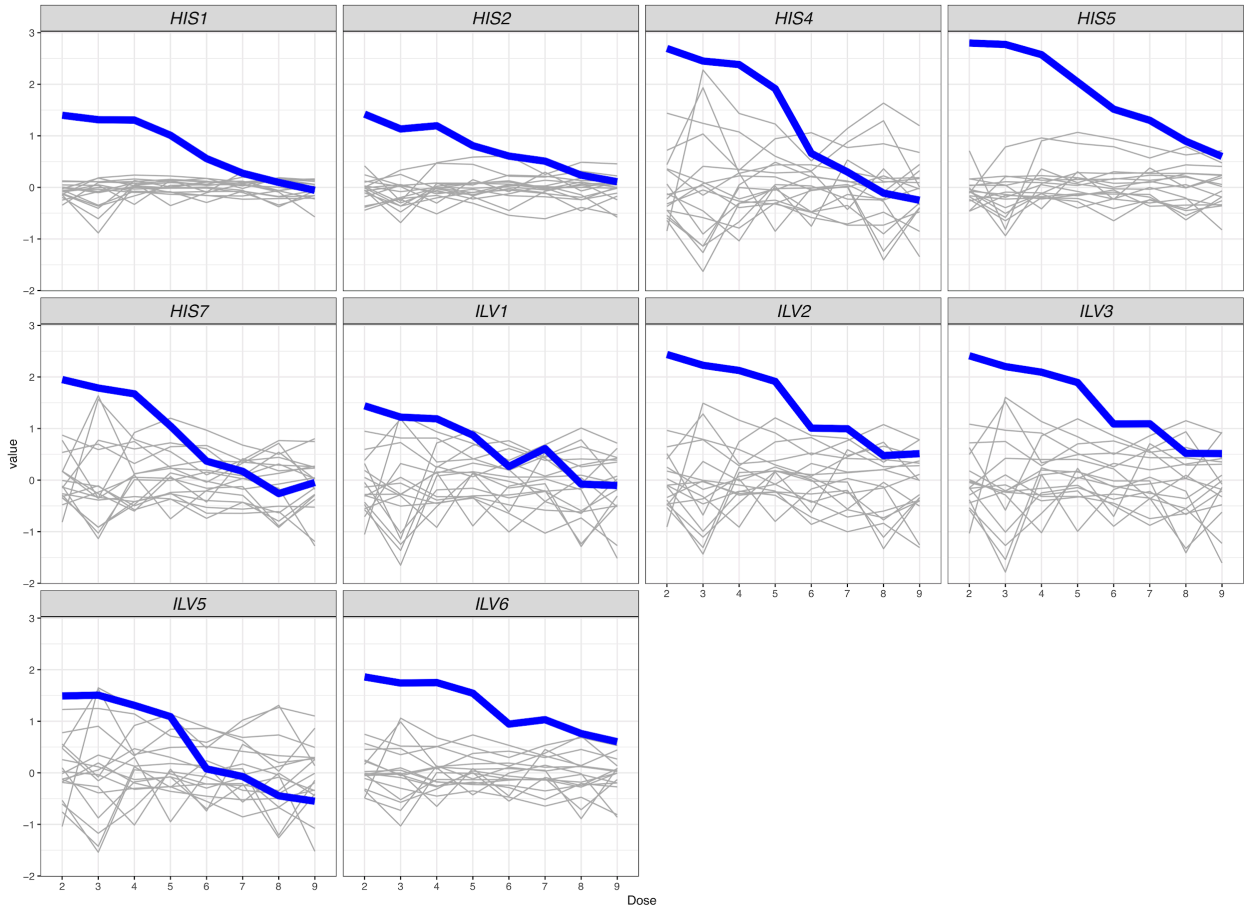

Appendix Figure S2: Gene expression responses of *ILV* and *HIS* genes with Bat1 dependence

TPM values for the highest eight doses were obtained for each genotype (labeled two through nine). For each gene, the TPM values were normalized to the median of TPM values for that gene across all samples, and then log2-transformed. Genes with Bat1-dependent repression were originally identified via hierarchical clustering. Data from the Bat1 induction experiment are displayed in blue. Data from other gene titration experiments are shown in grey.

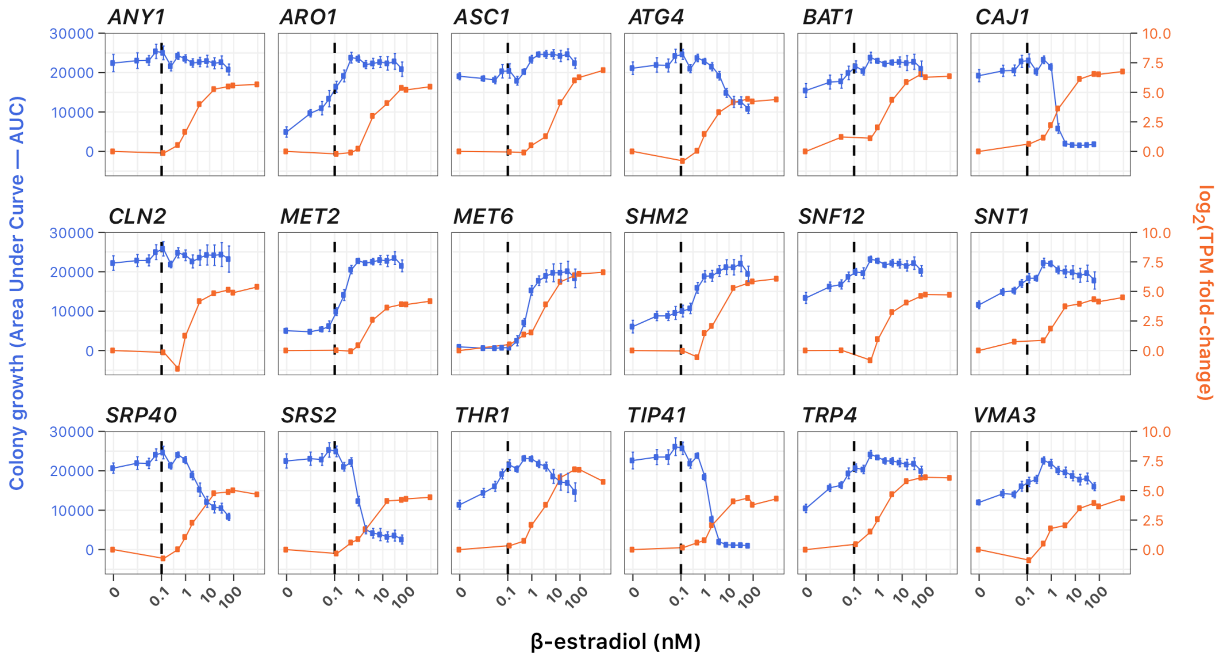

Appendix Figure S3: Comparing growth and transcriptional induction for 18 YETI-NE strains

Blue curves show growth data of 18 strains grown on minimal medium (on agar plates) with varying concentrations of β-estradiol. Error bars are +/- 1 standard deviation of the Area Under the Curve (AUGC) values acquired from eight independent colonies. Orange curves show log2 fold-change expression data of the same 18 strains from **Figure EV1A**. Dotted vertical grey lines are shown at 0.1 nM β-estradiol in each subplot.

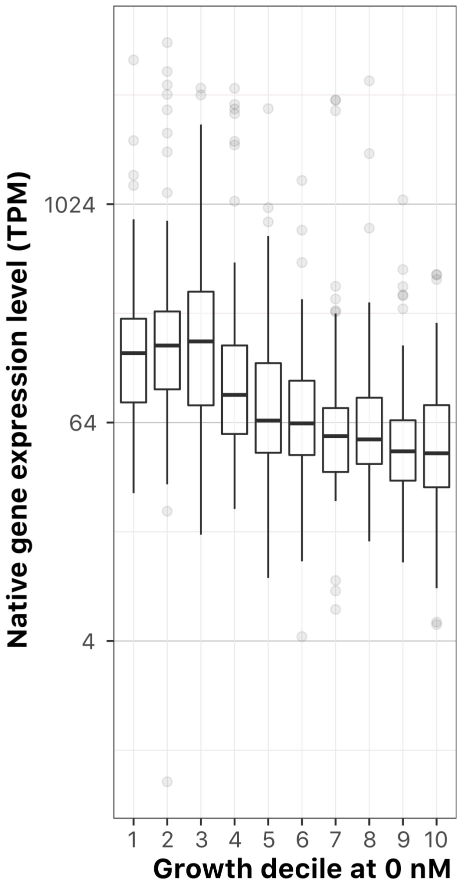

Appendix Figure S4: Native expression versus YETI strain growth without inducer

Box plot showing native levels of gene expression of genes from Lipson et al. (2009) (TPM, transcripts per million) as a function of the level of growth (from low to high, binned as growth deciles) of corresponding YETI-E strains at 0 nM β-estradiol.

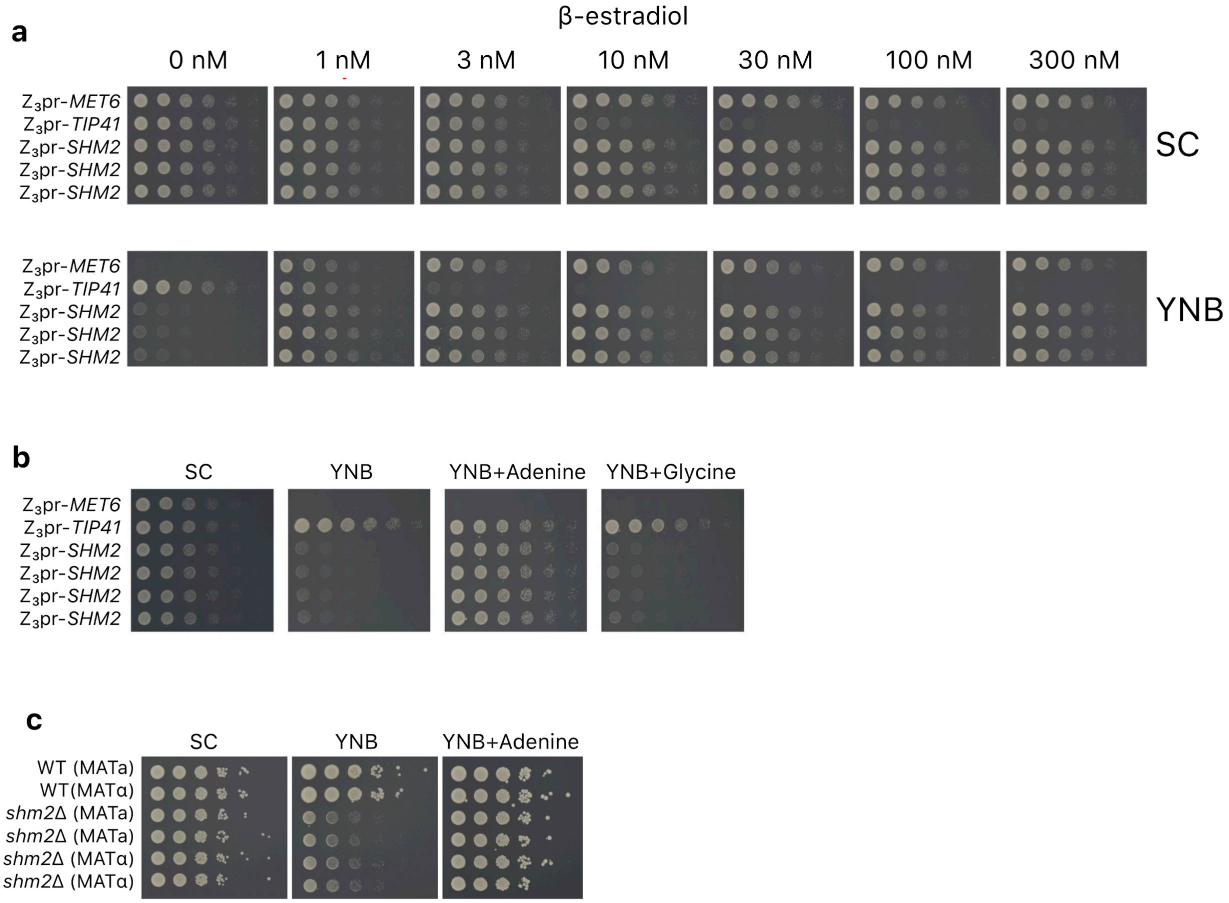

Appendix Figure S5: Z3pr-*SHM2* is an adenine auxotroph

A. Spot assays were performed with Z3pr-*MET6*, Z3pr-*TIP41*, and three independent colonies of Z3pr-*SHM2* on YNB and SC with increasing levels of β-estradiol. **B.** Spot assays were performed with Z3pr-*MET6*, Z3pr-*TIP41*, and four independent colonies of Z3pr-*SHM2* on SC, YNB, YNB with adenine, and YNB with glycine. **C.** Spot assays were performed with WT and four independent colonies of *shm2*Δ strains (two *MAT***a** and two MAT*α*) on SC, YNB, and YNB with adenine.

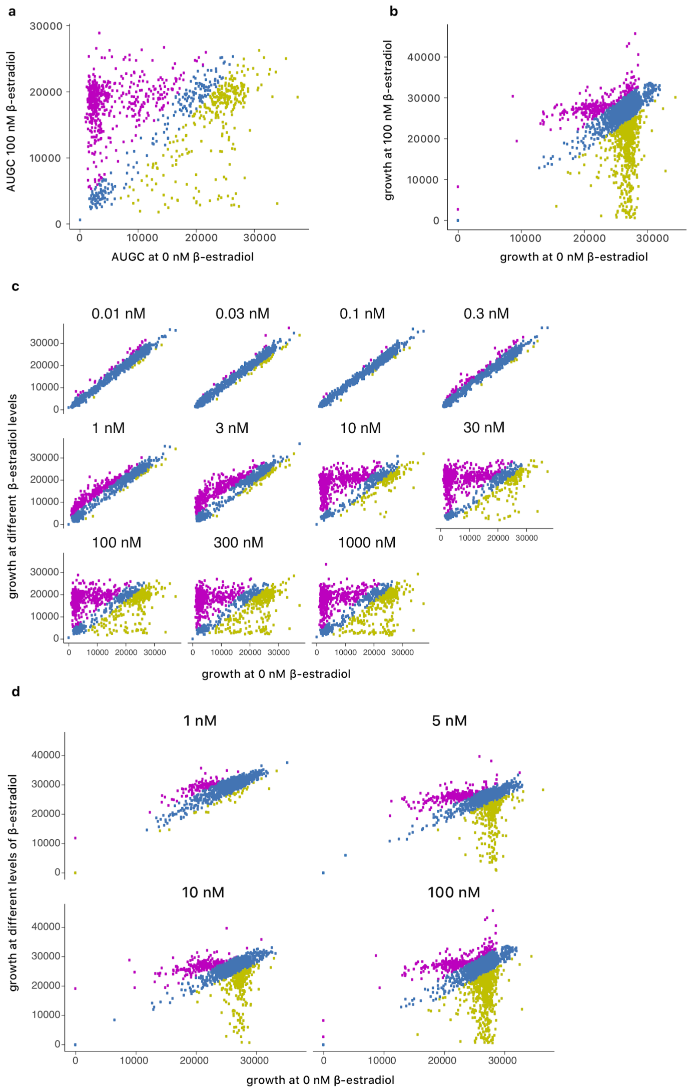

Appendix Figure S6: Identification of genes that impair growth when overexpressed

Growth (AUGC) in the presence (y-axis) and absence (x-axis) of β-estradiol for individual strains (points on the scatter plot). Blue points have a distance between -2,000 and 2,000 and are not considered dosage sensitive (Methods). Purple points have a distance less than -2,000 and yellow points have a distance greater than 2,000 and were identified as toxic strains. **A.** Toxic genes called for YETI-E strains grown at 100 nM and 0 nM. **B.** Toxic genes called for YETI-NE strains grown at 100 nM vs. 0 nM. **C.** Toxic genes called for YETI-E strains grown at β-estradiol concentrations (0.01, 0.03, 0.1, 0.3, 1, 3, 10, 30, 100, 300, 1000 nM) vs. 0 nM. **D.** Toxic genes called for YETI-NE strains grown at β-estradiol concentrations (1, 5, 10 and 100 nM vs. 0 nM).

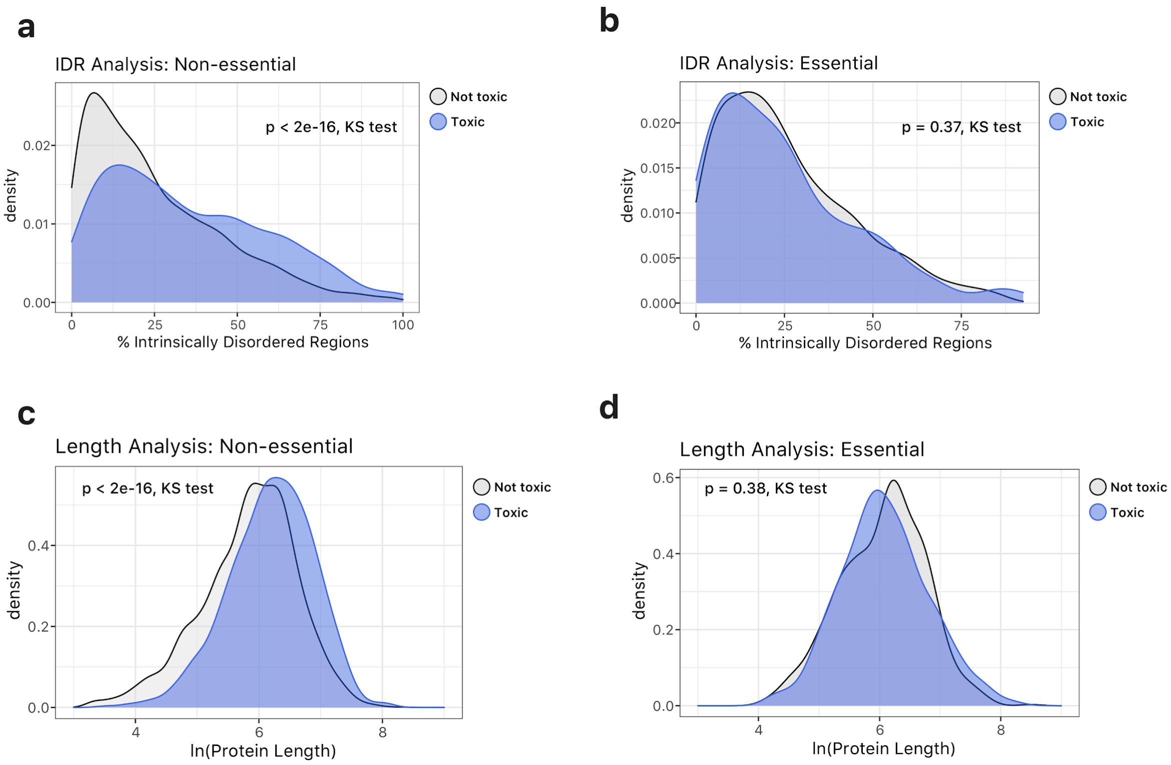

Appendix Figure S7: Non-essential toxic genes encode proteins that are bigger and more disordered than non-toxic genes

A. Density plots of YETI-NE genes identified as non-toxic/toxic when overexpressed based on the percent of the protein that belongs to an intrinsically disordered region. The sample sizes are 687 for toxic genes, and 3970 for non-toxic strains. **B.** Density plots of YETI-E genes identified as non-toxic/toxic when overexpressed based on the percent of the protein that belongs to an intrinsically disordered region. The sample sizes are 301 for toxic genes, and 721 for non-toxic strains. **C.** Density plots of YETI-NE genes identified as non-toxic/toxic when overexpressed based on protein length. **D.** Density plots of YETI-E genes identified as non-toxic/toxic when overexpressed based on protein length.

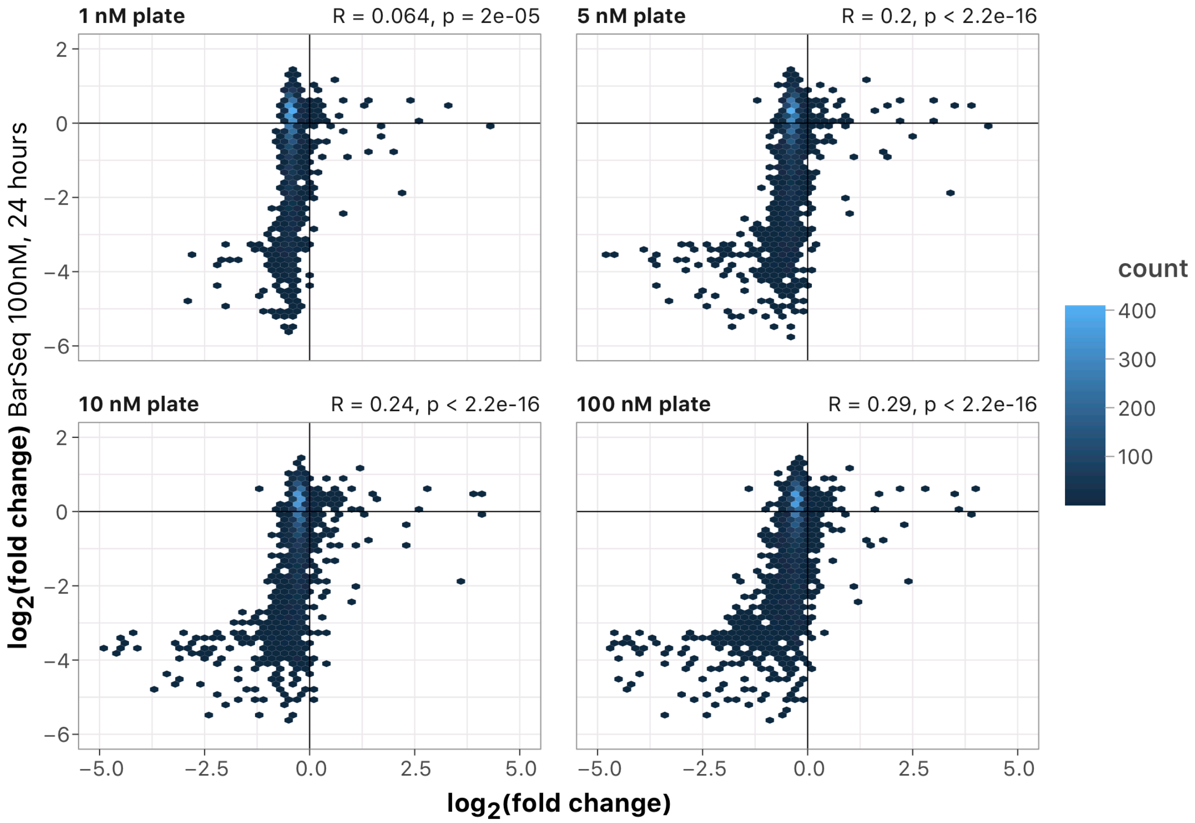

Appendix Figure S8: Comparing pooled screens to plate-based assays

Scatterplots of log2-fold-changes of barcode frequencies in pooled culture (y-axis) after 24 hours of growth versus log2-fold-changes in total growth of strains on plates. The Spearman coefficient, and its significance, is shown in red.

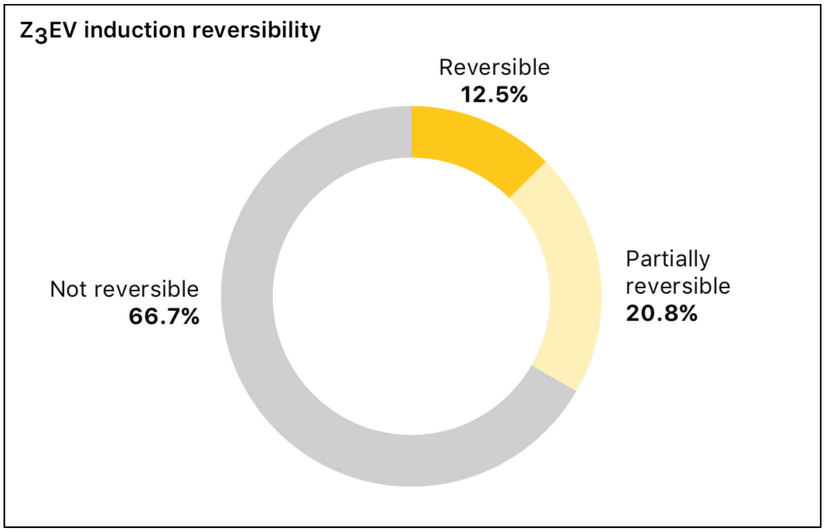

Appendix Figure S9: Conditional growth of Z_3EV-driven essential genes is not reversible in 16 of 24 strains tested

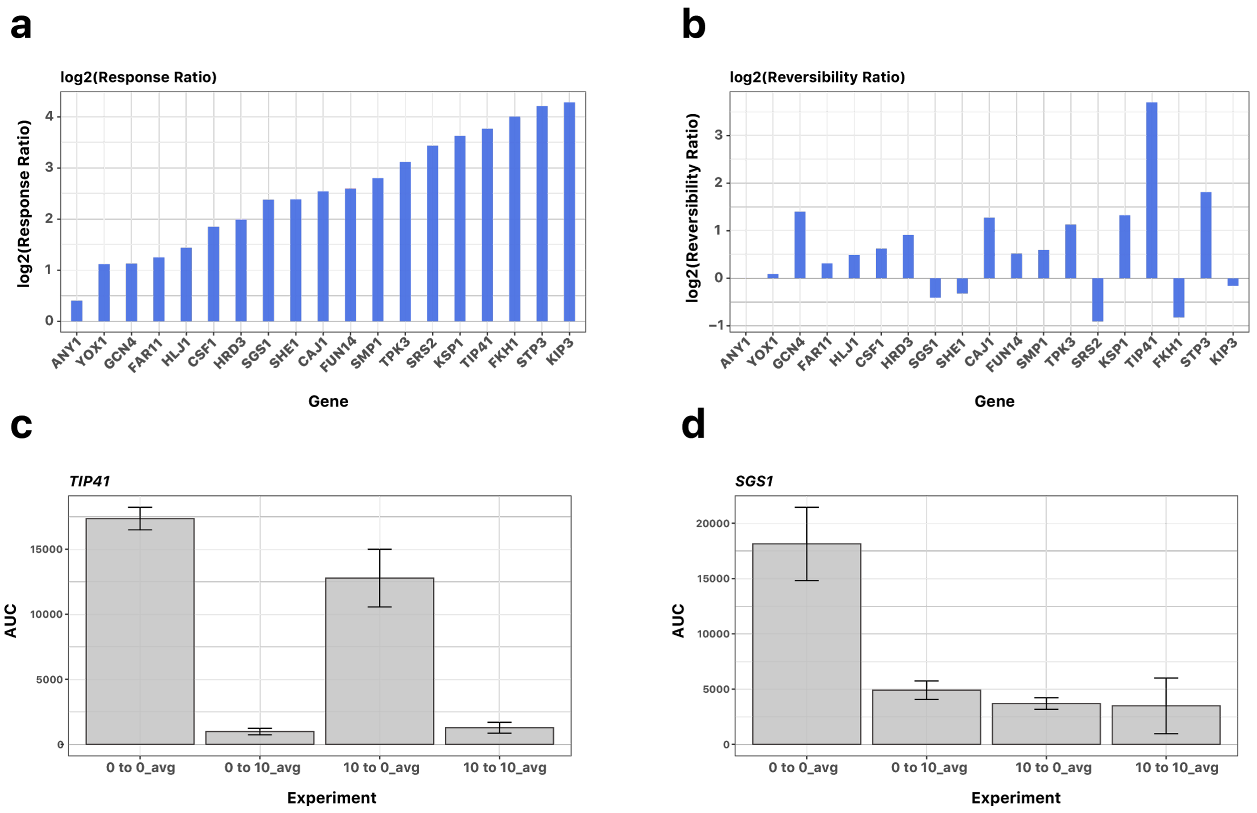

Appendix Figure S10: Reversibility analysis for 19 strains that impair growth when overexpressed

A. The Response Ratio, $ResRatio_i = AUGC_{i,0,0} / AUGC_{i,10,10}$, for each strain. For $AUGC_{i,j,k}$, *i* is the gene, *j* is the concentration of β-estradiol during the first round of growth, and *k* is the level of β-estradiol during the second round of growth. For a given gene, the larger the *ResRatio* value, the greater the toxicity when the gene is induced. If log2(*ResRatio*) is negative, the gene improves growth when induced. Genes are ranked from least toxic to most toxic when induced in the presence of β-estradiol **B.** The Reversibility Ratio, $RevRatio_i = AUGC_{i,10,0} / AUGC_{i,0,10i}$, for each strain. If $\log2(RevRatio_i) > 0$, the growth phenotype is at least partially reversible. **C-D.** Examples of reversible (C) and non-reversible (D) phenotypes. Barplots show total area under growth curve (AUGC) for cells grown at two β-estradiol concentrations and transferred to plates under four different conditions: 0 to 0_avg - a control transfer from no β-estradiol to no β-estradiol (no β-estradiol); 10 to 10_avg - another control transfer from β-estradiol to β-estradiol (continuous β-estradiol); 0 to 10_avg - transfer from no β-estradiol to β-estradiol (addition of β-estradiol) and; 10 to 0_avg - transfer from β-estradiol to no β-estradiol (removal of β-estradiol).

ATF	Zif268 binding sites	URS1 element	0	1	2	3	4	5	6	7	8	9	10	11	12	13	14	15	16	17	18	19	20	21	22	23	24	hours
Z3EV	6	-	44538	40273	40365	40092	41199	41379	40168	35806	31574	27519	22184	18225	14748	11677	9419	7643	6181	5194	4312	3715	3281	2880	2619	2455	2323	
Z3EV	2	-	9202	8245	8573	9249	9572	9365	8953	8157	7203	6191	5047	4182	3545	2919	2428	2001	1670	1427	1244	1077	975	890	816	780	755	
Z3EV	6	+	1913	1755	1583	1491	1486	1421	1349	1199	1060	902	762	671	578	498	434	400	361	336	314	299	286	278	271	269	269	
Z3EV	2	+	484	444	422	403	389	375	368	350	327	306	287	271	258	248	240	234	226	223	219	217	214	213	213	212	212	
Z3EB42	6	-	25163	23805	21675	19007	16812	13624	11239	8989	6975	5516	4401	3490	2803	2313	1809	1550	1315	1118	990	877	807	745	728	704	684	
Z3EB42	2	-	4484	4273	4296	4046	3871	3439	2890	2354	1931	1579	1287	1066	905	767	670	582	514	473	433	403	379	365	356	351	347	
Z3EB42	6	+	1051	928	837	734	653	573	497	429	383	345	321	295	277	261	252	241	234	231	226	222	219	216	214	214	214	
Z3EB42	2	+	335	318	308	292	284	273	262	248	241	234	228	222	220	217	215	212	210	211	209	207	208	207	203	204	206	
Z3EV	0	-	217	215	212	207	205	205	206	202	203	201	203	201	202	200	202	200	200	201	201	200	200	200	198	198	197	

Appendix Figure S11: Reversibility analysis of Z_3EV and Z_3EB42 using mNeonGreen reporter

Strains with all combinations of ATFs and promoters driving mNeonGreen were grown to early log phase in YNB + 1000 nM β-estradiol, washed 3x in PBS and resuspended in YNB without β-estradiol. Strains were imaged every hour using a Phenix automated confocal microscope and average GFP fluorescence/cell was calculated using Harmony software. Data are conditionally formatted such that high fluorescence is red and low fluorescence is blue. Numbers indicate arbitrary fluorescence units.
