# Supplementary material for: A genome‐scale yeast library with inducible expression of individual genes
Source: Mol Syst Biol. 2021 Jun 7;17(6):e10207. doi: 10.15252/msb.202110207 (PMC8182650; doi:10.15252/msb.202110207)
Supplement: Supplementary file 2 — Expanded View Figures PDF [file MSB-17-e10207-s013.pdf]

Expanded View Figures

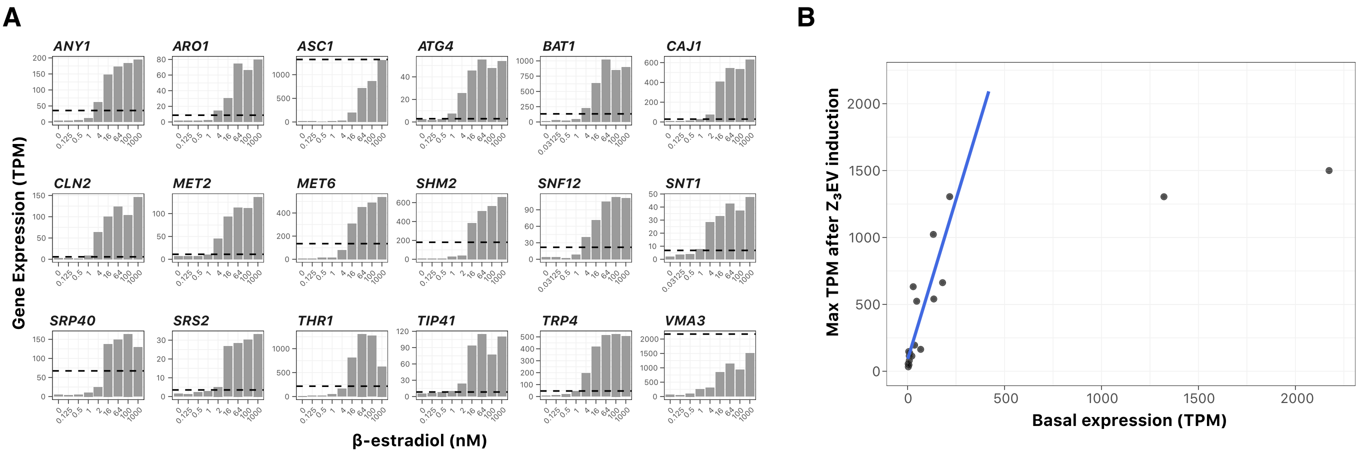

Figure EV1. Transcriptional induction of 18 YETI-NE strains.

A Expression data of 18 strains during exponential growth in 1 ml batch culture of SC medium after 30 min of induction with varying concentrations of β-estradiol in a deep-well 96-well plate. The *y*-axis shows transcripts per million (TPM) values from RNA-seq experiments, and the *x*-axis shows concentrations of β-estradiol (nM). The dotted line indicates the endogenous level of expression for each gene in this experiment, calculated as the average TPM value from all strains in the panel that contain a native copy of the allele.

B Comparing Z_3EV inducibility to endogenous gene expression. The graph shows maximum TPM values after β-estradiol induction for the 18 genes assayed in (A) (*y*-axis) versus native expression (*x*-axis). For genes with maximum endogenous expression level of < 250 TPM, a linear model was fit ($y = 87.96 + 4.79x$, $R^2 = 0.76$).

 © 2021 The Authors

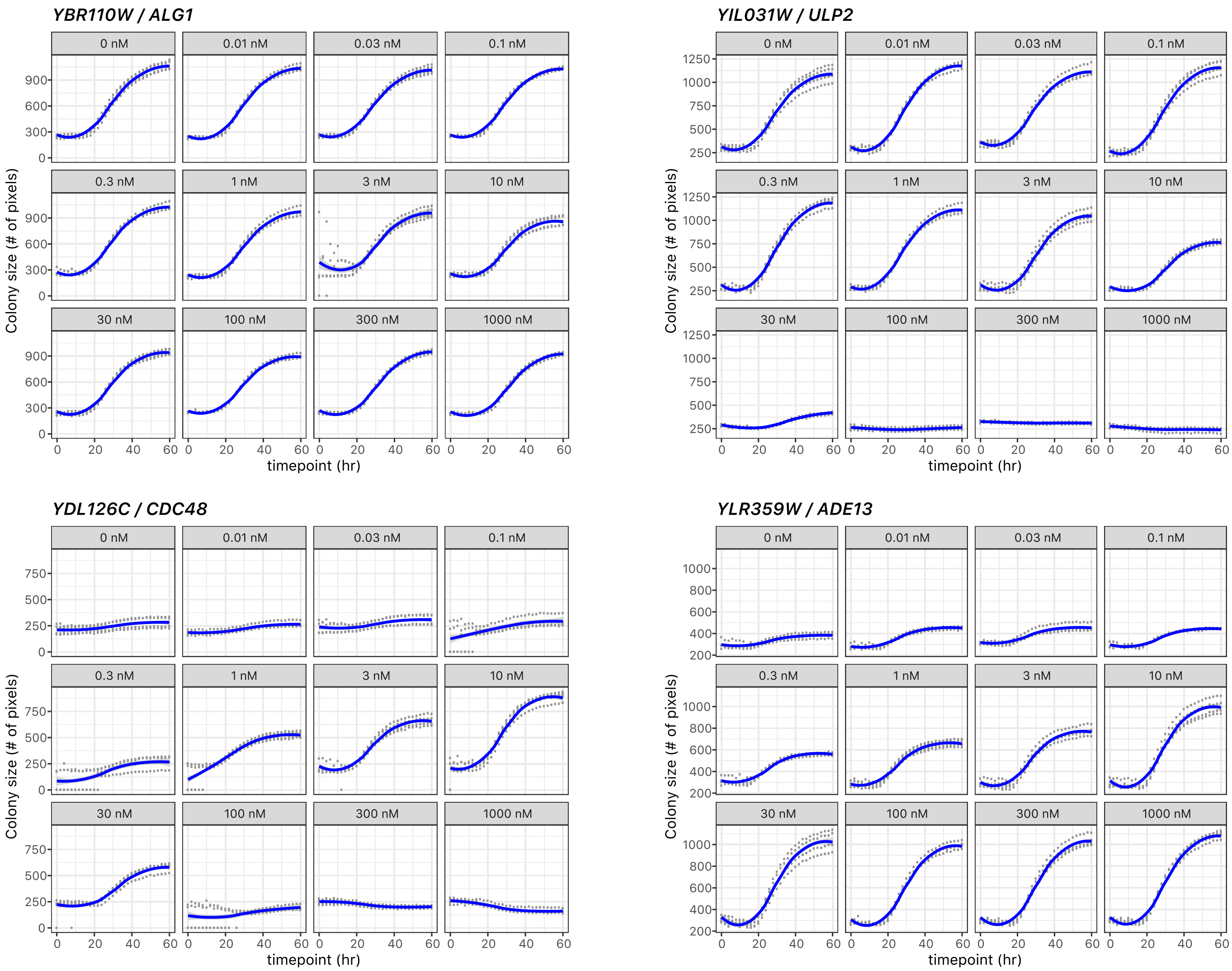

Figure EV2. Growth measurements for YETI alleles of ALG1, ULP2, CDC48, and ADE13.

LOESS curve fits are shown (blue lines) from individual measurements of four separate colonies (gray dots).

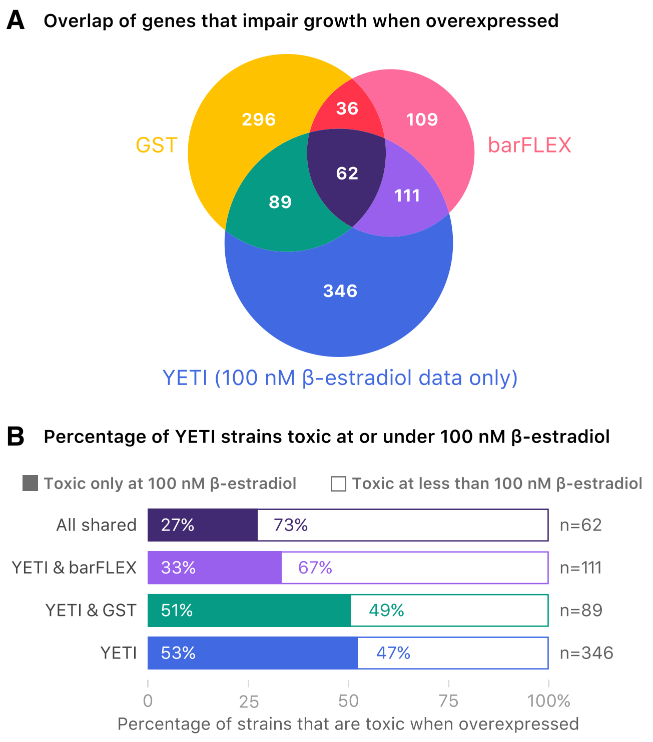

Figure EV3. Analysis of genes that impair growth when overexpressed.

A Venn diagram of genes that are toxic upon overexpression from this study as compared to previous overexpression studies.

B Barplot of Z_3EV toxic strains grouped by overlap with previous overexpression studies seen in (A). For each grouping the percentage of Z_3EV toxic strains that are toxic at only 100 nM β-estradiol (solid color) versus the percentage that are toxic at β-estradiol concentrations < 100 nM (white) is shown.

 © 2021 The Authors

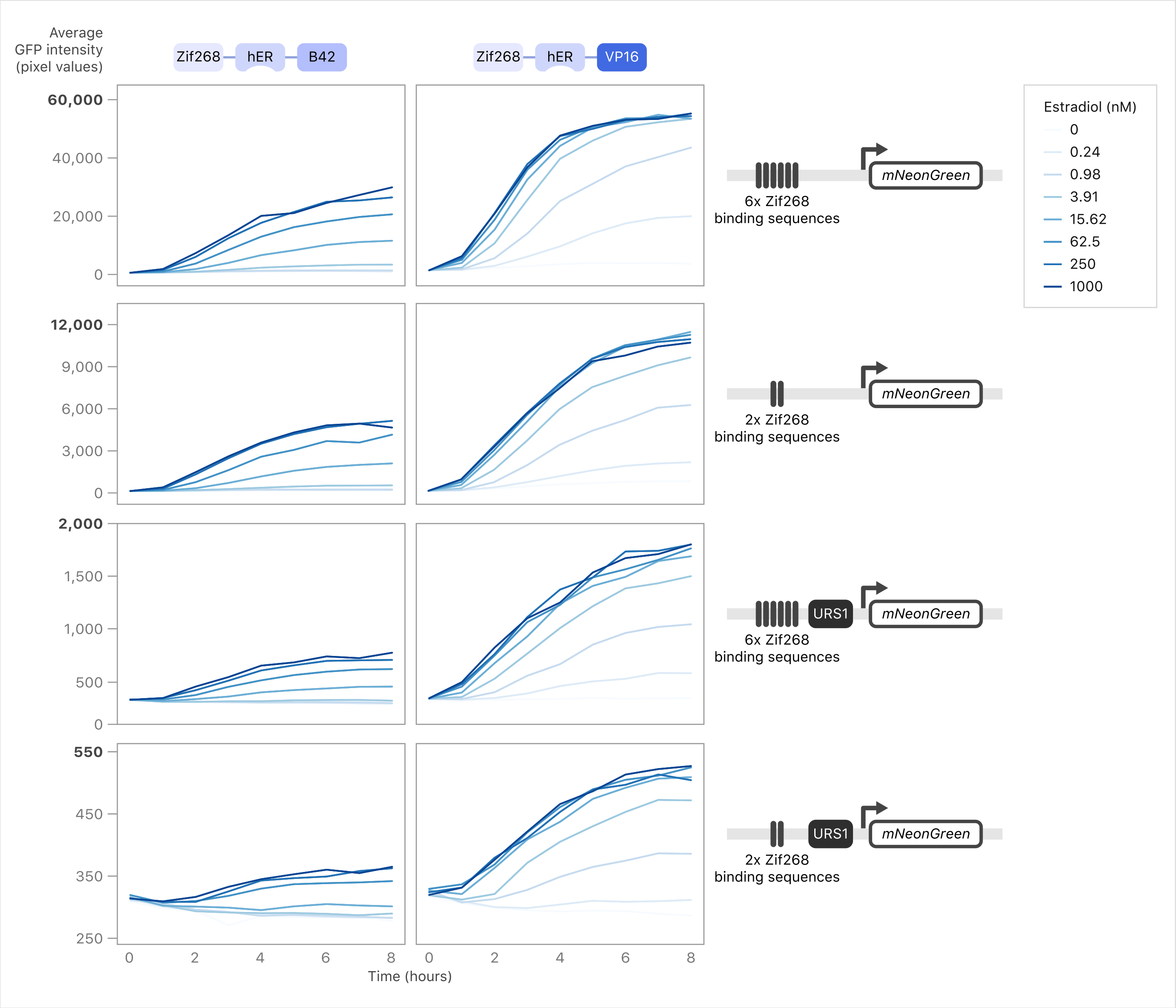

Figure EV4. Number of binding sites, URS1 presence, and ATF choice all affect mNeonGreen reporter induction time series.

Full time series collected for testing the relationship between ATF, URS1, and binding site copy number. Strains were imaged every hour using a Phenix automated confocal microscope, and average fluorescence/cell was calculated using Harmony software. Average of two replicates is shown following induction with > 300 cells quantified per dose per replicate.

© 2021 The Authors

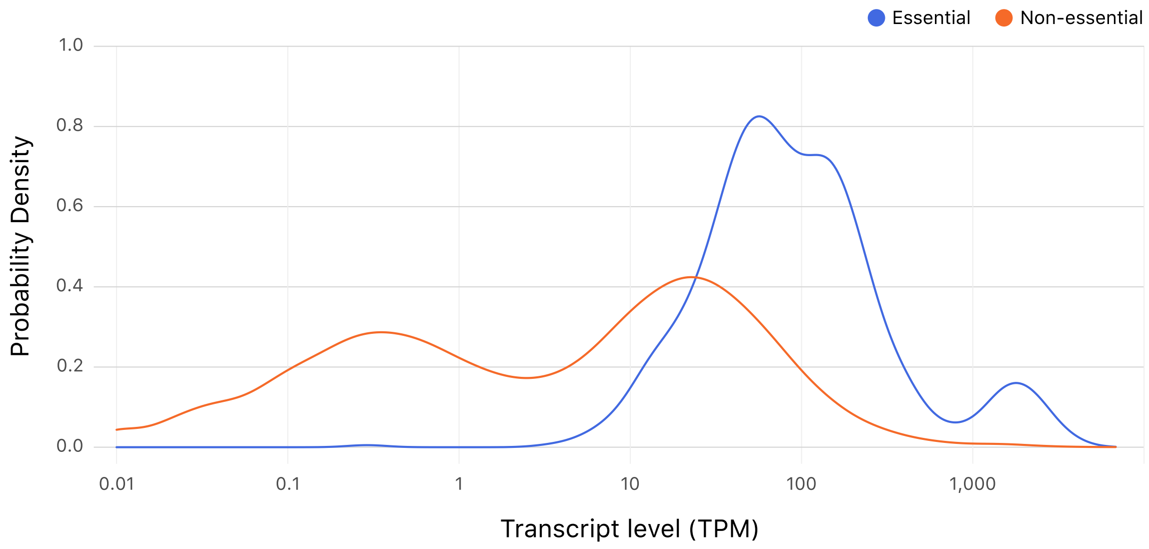

Figure EV5. Expression (TPM) levels of Core Essential Genes (CEG2) Essential/Non-Essential genes in K562 cells.

The CEG2 essential list is from (Hart *et al*, 2017). RNA-seq data can be found on the Gene Expression Omnibus with accession GSE88351. The median TPM of CEG2 non-essential genes is 5 TPM. The median TPM of CEG2 essential genes is 80 TPM.

 © 2021 The Authors
