# Supplementary material for: A genome‐scale yeast library with inducible expression of individual genes
Source: Mol Syst Biol. 2021 Jun 7;17(6):e10207. doi: 10.15252/msb.202110207 (PMC8182650; doi:10.15252/msb.202110207)
Supplement: Supplementary file 13 — Dataset EV11 [file MSB-17-e10207-s015.zip › MSB-2021-10207RR-Dataset_EV11/Dataset EV11 Legend.docx]

**Dataset EV11: BAR-seq experiment .CDT and .GTR files.**
