# Supplementary material for: A genome‐scale yeast library with inducible expression of individual genes
Source: Mol Syst Biol. 2021 Jun 7;17(6):e10207. doi: 10.15252/msb.202110207 (PMC8182650; doi:10.15252/msb.202110207)
Supplement: Supplementary file 14 — Dataset EV12 [file MSB-17-e10207-s006.zip › MSB-2021-10207RR-Dataset_EV12/Dataset EV12 Legend.docx]

**Dataset EV12: URA3-Z_3_pr sequence**
